# Selenium is a modulator of circadian clock that protects mice from the toxicity of a chemotherapeutic drug via upregulation of the core clock protein, BMAL1

**DOI:** 10.18632/oncotarget.411

**Published:** 2011-12-31

**Authors:** Yan Hu, Mary L. Spengler, Karen K. Kuropatwinski, Maria Comas, Marilyn Jackson, Mikhail V. Chernov, Anatoly S. Gleiberman, Natalia Fedtsova, Youcef M. Rustum, Andrei V. Gudkov, Marina P. Antoch

**Affiliations:** ^1^ Department of Molecular and Cellular Biology, Roswell Park Cancer Institute, Buffalo, NY; ^2^ Department of Cell Stress Biology, Roswell Park Cancer Institute, Buffalo, NY; ^3^ Small Molecule Screening Core Facility, Roswell Park Cancer Institute, Buffalo, NY; ^4^ Cleveland BioLabs, Inc, Buffalo, NY; ^5^ Department of Cancer Biology, Roswell Park Cancer Institute, Buffalo, NY

**Keywords:** L-Methyl-selenocysteine, TIEG1, circadian rhythms, cyclophosphamide, anticancer treatment, liver, transcription

## Abstract

Selenium compounds are known as cancer preventive agents and are also able to ameliorate the toxicity associated with anti-cancer radiation and chemotherapy in mouse models. Sensitivity to the toxicity of chemotherapy is also modulated by the circadian clock, molecular time-keeping system that underlie daily fluctuations in multiple physiological and biochemical processes. Here we show that these two mechanisms are interconnected. By screening a library of small molecules in a cell-based reporter system, we identified L-methyl-selenocysteine as a positive regulator of the core clock protein, BMAL1. L-methyl-selenocysteine up-regulates BMAL1 at the transcriptional level both in cultured cells and in mice. We also show that in tissue culture selenium exerts its action by interfering with TIEG1-mediated repression of *Bmal1* promoter. Selenium treatment fails to protect BMAL1-deficient mice from toxicity induced by the chemotherapeutic agent cyclophosphamide but does protect *Clock* mutant mice deficient in circadian rhythm control but having normal BMAL1. These findings define selenium as circadian modulator and indicate that the tissue protective effect of selenium results, at least in part, from up-regulation of BMAL1 expression and subsequent enhancement of CLOCK/BMAL1-mediated transcription.

## INTRODUCTION

Selenium is an essential trace element of fundamental importance to human health due to its anti-oxidant, anti-inflammatory and anti-viral activities [1]. Various forms of selenium (both organic and inorganic) have been shown to have chemopreventive effects in a variety of malignancies, including breast and prostate cancer [2, 3]. An organic form of selenium, L-methyl-selenocysteine (MSC), acts as a selective modulator of the therapeutic efficacy and systemic toxicity of anticancer drugs in a variety of human tumor xenograft models [4-6].

The molecular mechanisms of selenium action have been extensively investigated and multiple mechanisms have been proposed, suggesting that the biological effects of this compound are likely highly pleiotropic. Traditionally, many aspects of selenium action have been attributed to its anti-oxidant activity. In the form of selenocysteine, it is incorporated into several anti-oxidant enzymes, such as glutathione peroxidases and thioredoxin reductases, which are important for regulating the balance of reactive oxygen species (ROS) in an organism [7]. More recently, several aspects of selenium action have been linked to both positive and negative regulation of transcription factors that are involved in control of normal cellular proliferation and cellular responses to stress conditions, such as NFκB, Sp1 and Sp3 [8], HIF-1α [4] and p53 [9]. Taken together, these data imply that selenium may modulate various pathways and that this regulation is likely to be tissue-specific.

Many aspects of mammalian physiology are controlled by the activity of components of the circadian clock (reviewed in [10]. This evolutionarily conserved system is comprised of a network of transcriptional and translational feedback loops that drive 24-hr-based oscillations in RNA and protein abundance of key clock components [11, 12]. This, in turn, translates into 24-hr periodicity in expression of a large number of output genes [13]. The molecular mechanism of circadian oscillation is operative in virtually all mammalian tissues and thereby influences a wide range of physiological and metabolic processes in a tissue-specific manner [14].

One of the important processes that are modulated by the circadian clock is an organism’s response to genotoxic stress, such as that induced by anticancer drug and radiation treatments. Previously, using circadian mice deficient in different components of the circadian clock, we established a molecular link between the functional status of the major circadian CLOCK/BMAL1 transcriptional complex and the sensitivity of normal tissues to toxicity induced by the chemotherapeutic agent cyclophosphamide (CY) [15]. These data, together with our more recent work [16, 17], allowed us to define circadian transcriptional activators as potential targets for pharmacological modulation aimed at protecting normal tissues from damage induced by genotoxic treatments.

Here we report that selenium up-regulates the core circadian protein, BMAL1, by relieving the GC-box/TIEG1-mediated repression of *Bmal1* promoter The *in vivo* effect of selenium was found to be tissue-specific in that selenium-induced changes in BMAL1 were detected in the liver, but not in the suprachiasmatic nucleus (SCN), the central pacemaker of the circadian system. Consistent with this tissue-specificity, systemic administration of selenium did not affect circadian behavioral parameters; however, it did result in a significant increase in animals’ resistance to toxicity induced by the chemotherapeutic agent, cyclophosphamide. These data reveal novel mechanisms of selenium action that are linked to regulation of the core clock components.

## RESULTS

### A cell-based readout system reveals selenium compound as a circadian clock modulator

Our previous studies identified CLOCK/BMAL1 transcriptional activity as a target for pharmacological intervention to, among other potential applications, reduce the toxicity associated with genotoxic anti-cancer treatments [16]. To identify small molecules capable of modulating CLOCK/BMAL1 activity, we designed a readout system based on mouse fibrosarcoma L929 cells expressing high levels of endogenous CLOCK and BMAL1, positive regulators circadian transcriptional machinery, transduced with *Per1*-driven luciferase reporter construct (L929-*Per1*) [18]. By screening a chemical library of known pharmacological agents (LOPAC, Sigma, 1280 compounds total) in this system, we identified 14 inhibitors and 17 activators of *Per1* promoter as our major hits (Supplementary Tables 1 and 2).

**Figure 1:**
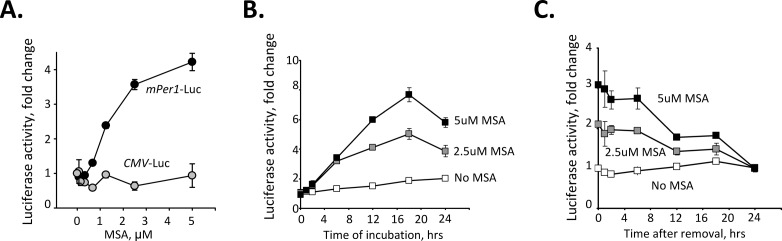
Selenium specifically activates circadian promoter in a time and dose-dependent manner (A). Selenium activates *Per1* promoter in a dose-dependent manner. L929 cells carrying luciferase reporter genes under the control of either *mPer1* or CMV promoters were treated with the indicated concentrations of MSA for 18 hrs. Luciferase activity was measured in cell lysates and normalized for β-Gal expression. The experiment was performed in triplicate; the values presented are mean fold-change relative to that in untreated cells ± standard error. (B). Selenium activates *Per1* promoter in a time-dependent manner. L929-*Per1*-Luc cells were treated with different concentrations of MSA (0, 2, 5μM). Cells were collected at various time points over a period of 36 hrs and luciferase activity was measured. (C) L929-*Per1*-Luc cells were pretreated with different concentrations of MSA (0, 2, 5μM) for 18 hrs. MSA was then washed out and cells were collected at various time points over a period of 24 hr for measurement of luciferase activity. Values are mean from triplicates with error bars indicating standard deviation.

Among our primary hits, there were several known regulators of circadian function such as glucocorticoids [19], 2-methoxyestradiol [20], forskolin [21], PKC and p38 MAPK inhibitors [22, 23], which validated the feasibility of our screening approach. Other compounds from this list, however, have not been previously linked to circadian function. Thus, among the chemicals identified as potent upregulators of *Per1* promoter we were intrigued to find an organic selenium compound, L-methyl selenocysteine (MSC). Subsequent experiments using methylseleninic acid (MSA, a form of MSC optimized for *in vitro* studies) showed that treatment with MSA specifically induced an increase in *Per1*-driven luciferase activity in a dose-dependent manner (Fig. 1A). A significant increase in reporter signal can be detected as early as 6 hrs after MSA treatment reaching its maximum at 18 hrs post incubation (Fig. 1B). This promoter induction is reversible, as after removal of MSA, activity of *Per1*-driven luciferase remained high for ~6 hrs, after which it returned to its basal level within ~ 24 hrs (Fig. 1C).

**Figure 2:**
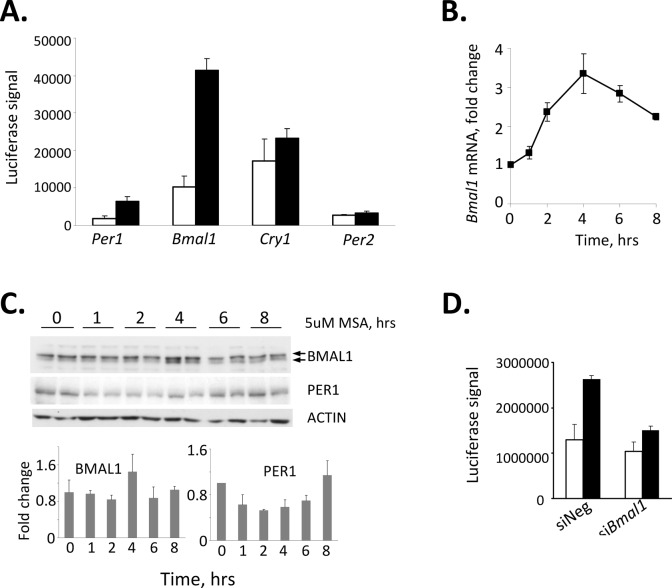
Selenium-dependent up-regulation of the *Per1* promoter is mediated through BMAL1 (A) Treatment with selenium results in acute induction of the *Bmal1* promoter. 293T cells were transfected with indicated reporters; 24hrs after transfection cells were treated with 5uM MSA for 6 hrs and luciferase activity was measured in cell lysates. Values represent mean from triplicates with error bars indicating standard deviation. (B) Treatment with selenium results in an increase of endogenous *Bmal1* mRNA. L929 cells were treated with 5uM of MSA for indicated times. The relative abundance of *Bmal1* mRNA was determined by real-time RT-PCR using the comparative delta Ct method. The final measurements were normalized based on *Gapdh* mRNA expression and are shown as the fold-change. (C) Selenium increases the abundance of BMAL1 protein. L929 cells were treated with 5uM of MSA for indicated times. The abundance of BMAL1 and PER1 in whole cell extracts was analyzed by Western blot (top panel). Two individual samples for each time point are shown. Lower panel presents quantitative analysis of BMAL1 and PER1 expression showing an increase in BMAL1 in 4hrs after MSA treatment followed by an increase in PER1. (D) Suppression of BMAL1 by specific siRNA abrogates selenium-mediated increase in *Per1* activation. L929 cells containing the *Per1*-Luc construct were transfected with siRNA against BMAL1 or control non-targeting (-) siRNA. 72hrs post-transfection, cells were treated with 5μM MSA and luciferase activity was measured in cell lysates. The values represent mean ± standard deviation.

The rapid kinetics of induction of luciferase activity by MSA suggested that selenium may exert its effect at the level of transcriptional activation. To determine which of the clock genes could be targeted by selenium, we tested the affect of MSA on basal promoter activity of *Clock*, *Bmal1*, *Per1*, *Per2* and *Cry1* genes using a luciferase reporter assay. Of all the promoters tested, only *Per1* and *Bmal1* were up-regulated by MSA, with the *Bmal1* promoter showing the highest fold of activation (Fig. 2A). MSA-induced activation of *Bmal1* gene transcription was confirmed by measuring levels of corresponding endogenous *Bmal1* mRNA in L929 cells at different times after MSA treatment (Fig. 2B). The peak time of RNA expression (4 hrs after incubation with MSA) is tightly coupled with increase in BMAL1 protein (Fig. 2C), whereas kinetics of modest increase in PER1 protein suggests that it is dependent on the activation of BMAL1 (Fig. 2C). Consistent with this, suppression of BMAL1 by specific siRNA completely blocked the stimulatory effect of MSA on CLOCK/BMAL1-driven activation of the *Per1* promoter (Fig.2D). The abundance of other clock transcripts and proteins including CLOCK, PER2, CRY1 and CRY2 was not affected by MSA (data not shown). Together, these data suggest that MSA-induced activation of the *Per1* promoter results from up-regulating the *Bmal1* gene followed by an increase in abundance and presumably activity of the corresponding protein.

### The effects of selenium on BMAL1 are mediated through transcriptional repressor TIEG1

**Figure 3:**
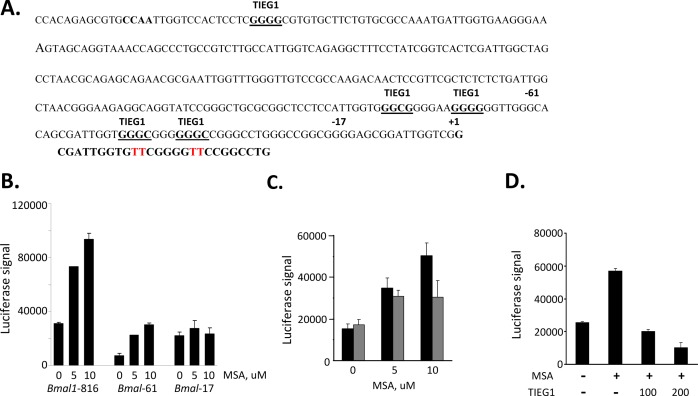
Selenium mediates *Bmal1* upregulation through TIEG1 repressor binding sites (A) Nucleotide sequence of the putative core promoter of the *mBmal1* gene. The transcription start site of ORF is designated +1. Potential TIEG1 binding sites are underlined. The *Bmal1* promoter fragments used (*Bmal1*-61 and *Bmal1*-17) are indicated by bold number above the truncation sites (-17 and -61). (B). Selenium up-regulates *Bmal1* promoters that contain the most proximal TIEG1 binding-sites. 293T cells were transfected with one of three *Bmal1* promoter fragmented reporter genes (816bp, 61bp, or 17bp upstream from +1bp transcription start site). 18hour post transfection cells were treated with or without indicated concentrations of selenium (MSA) and harvested 9 hours later to be analyzed for luciferase expression. (C). Selenium-mediated up-regulation of *Bmal1* gene is compromised in a promoter lacking the two most proximal TIEG1 sites. *Bmal1*-816 was used as a template to generate a promoter lacking the two proximal TIEG1-sites, which were previously determined to be responsible for *Bmal1* gene repression [32]. The primer used in the site-directed mutagenesis is shown below the two TIEG1 proximal sites, and the specific mutated base pairs are shown in red. Cells were transfected with either wild-type *Bmal1*-816 reporter gene (black bars) or TIEG1 binding site mutant *Bmal1*-816 (gray bars) and treated without or with MSA at 5 and 10uM.

Since our results in tissue culture demonstrated that MSA activates the *Bmal1* promoter, we sought to determine the region important for this regulation. It has been previously reported, that *Bmal1* is regulated by two transcription factors REV-ERBα (transcription repressor) and RORα (transcription activator) competing for the same RORE element in its promoter region [24-27]. More recently, the glucose-inducible gene *Tieg1*, which encodes an Sp1 family transcription repressor, was identified as another important regulator of *Bmal1* transcription. The Sp1 and Sp1-like transcription factors have been linked to the regulation of several genes by selenium [28-30]. The mechanism of selenium regulation has not been elucidated, but is likely to involve regulation of zinc-finger DNA binding. Previous analysis of the *Bmal1* promoter revealed three potential Sp1 binding sites in proximity to the transcription initiation site [31]; and of these three sites, the two most proximal Sp1-binding sites were shown to bind TIEG1 and repress *Bmal1* gene promoter [32](Fig. 3A). To test whether selenium might inhibit TIEG1 binding to these proximal Sp1-binding sites, truncated *Bmal1* promoters that included the two most proximal TIEG1-binding sites (*Bmal1*-816 and *Bmal1*-61) and one that excluded TIEG-binding site (*Bmal1*-17) were used in a luciferase transcription assay. MSA was shown to upregulate *Bmal1* promoters that included the proximal TIEG1-sites and MSA effect was ablated with a *Bmal1* promoter lacking the proximal TIEG1 sites (Fig. 3B). To further confirm the importance of the proximal TIEG1 repressor binding sites in MSA effect on *Bmal1* expression, the *Bmal1*-816 plasmid construct was used as a template in site-directed mutagenesis to generate specific point mutations of the proximal TIEG1 sites using the primer depicted in Figure 3A. The *Bmal1* promoter lacking the two most proximal TIEG1 sites was compromised for MSA-induction (Fig. 3C). We next asked whether the circadian regulated TIEG1 might overcome the upregulation of *Bmal1* promoter by MSA. Cells were co-transfected with *Bmal1*-816bp reporter gene and empty vector or increasing amounts of TIEG1 expression plasmid. The transcriptional luciferase results clearly show that high levels of ectopic TIEG1 represses *Bmal1* expression even in the presence of MSA (Fig. 3D). These data suggest that selenium mediates its effects on *Bmal1* expression at least in part through the two most proximal TIEG1 elements.

### Selenium administration increases the abundance of the BMAL1 protein *in vivo*

**Figure 4:**
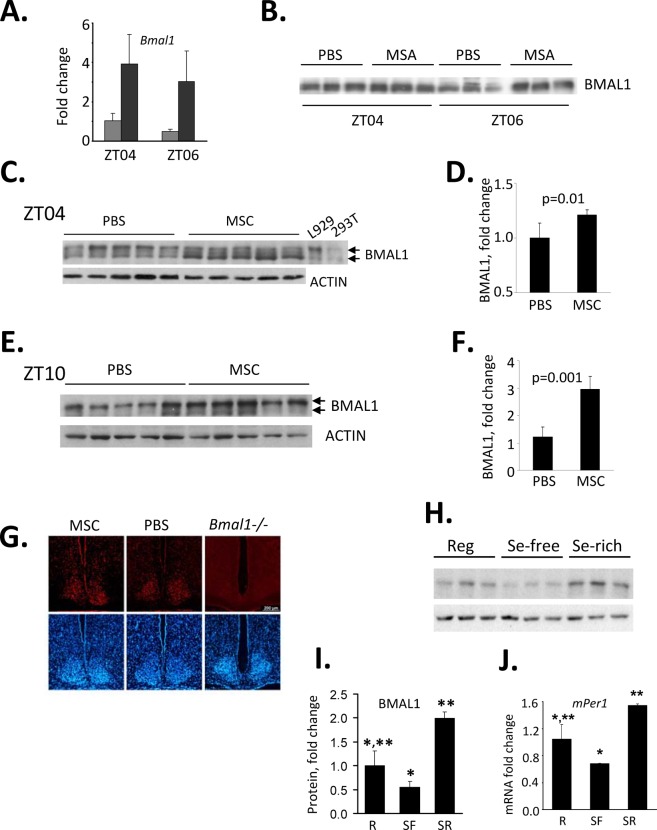
Selenium up-regulates *Bmal1* expression in mouse liver (A) Selenium administration results in acute induction of *Bmal1* transcript in mouse liver. C57BL/6J mice received singe i.p. injection of MSC (10mg/kg) or PBS at ZT03 (zeitgeber time, ZT00 corresponds to the time of lights on). Livers were collected 1 and 3 hrs later (ZT04 and ZT06 respectively), and relative abundance of *Bmal1* mRNA was measured by real-time RT-PCR or PBS at ZT03 (zeitgeber time, ZT00 corresponds to the time of lights on). Data present mean values for three mice ± standard deviation. (B) Selenium-induced activation of the *Bmal1* promoter results in increase in BMAL1 protein in liver. Western blot analysis of total lysates of liver obtained from the same animals as in (A). (C-F) Selenium increases BMAL1 protein in liver when administered by oral gavage. Western blots (C,E) and their quantitative analysis (D,F). Twenty animals received either PBS or MSC (7.5 mg/kg, oral gavage) daily for two weeks. Tissue samples were collected on day 15 at either ZT04 (C,D) or ZT10 (E,F) and probed with anti-BMAL1 antibody. Each lane represents tissue lysate obtained from an individual animal. Actin was used to control for loading. ImageQuant software was used for Western blots quantitation. Cell lysates from L929 and 293T cells were used as positive controls for endogenous and ectopically expressed BMAL1 respectively. (G) Selenium has no effect on BMAL1 expression in the SCN. Animals were fed daily with either PBS or MSC (7.5 mg/kg, oral gavage) for two weeks. Animals were then sacrificed for preparation of SCN tissue sections. The 12 μm sections were stained with anti-BMAL1 antibody followed by staining with a fluorescently-labeled secondary antibody (red). DAPI (blue) was used to stain the nuclei of all cells. SCN sections obtained from untreated *Bmal1−/−* mice are shown as controls for specificity of the antibody. Consistent with previous reports [52], no daily variations in BMAL1 immuno-reactivity were detected. Representative sections of the brains collected at ZT02 are shown. (H) The abundance of BMAL1 protein in mouse livers correlates with the amount of selenium in their diets. Animals were fed regular (Reg), selenium-free (SF) or selenium-enriched (SR) diets for 6 weeks. Tissue samples were collected at ZT10 and used for Western blotting with anti-BMAL1 antibody. Each lane represents tissue lysate obtained from an individual animal. (I) Quantitation of the Western blot (G) using ImageQuant software. Expression of BMAL1 was normalized based on ACTIN levels. Values represent mean ± standard error, n=3/group. *p=0.06; **p=0.007 (Student’s t-test). (J) Selenium-induced changes in the level of BMAL1 correlate with increased level of its direct transcriptional target *Per1* in the liver. Same animals as in G were used. Relative mRNA abundance was determined by real-time RT-PCR using the comparative delta Ct method. The final measurements were normalized by *Gapdh* mRNA expression. Values represent mean ± standard deviation, n=3/group. *p=0.04; **p=0.01 (Student’s t-test).

To test whether selenium has a similar stimulatory effect and kinetics on BMAL1 protein *in vivo* as observed in culture, we administered it in the form of MSC to mice as a single i.p. injection (10 mg/kg). The injection was performed at ZT03, when *Bmal1* expression is normally at its lower daily levels and livers were collected 1hr and 3hrs post injection. Similar to our results obtained in tissue culture, selenium administration caused a significant increase in *Bmal1* RNA (Fig. 4A) and protein (Fig. 4B).

Next, we tested whether this increase could be induced by prolonged systemic administration of selenium. For this, we administered MSC via gavage daily for a period of 2 weeks, and measured levels of BMAL1 protein in two tissues: the liver, which is the major metabolic tissue with prominent circadian regulation, and in neurons of the SCN, the site of the mammalian master clock. To account for normal circadian variations in BMAL1 levels in the liver, tissue samples were collected at two time points, ZT04 and ZT10. Similar to acute induction in response to a single administration, systemic administration caused a significant increase in BMAL1 protein levels in the liver at both times tested (Fig. 4C-F). At the same time, despite the ability of selenium to cross the blood-brain barrier [33], no increase in BMAL1 immunoreactivity was detected in the SCN of animals fed with MSC compared to animals fed with PBS (Fig. 4G) independent of time of brain collection.

The BMAL1 abundance in the liver directly correlated with the amount of selenium when it was administered as a dietary supplement in the form of sodium selenite (Fig. 4H,I). These diet-associated differences correlated with the levels of *Per1* mRNA expression measured at its normal peak time at ZT10 (Fig. 4J). Consistent with the lack of selenium effect on the abundance of BMAL1 in the SCN, circadian behavioral parameters were not affected by the type of diet (Fig. S1, Table S3). These data suggest that selenium affects the level and activity of BMAL1 in a tissue-specific manner and that selenium-mediated regulation of BMAL1 may play an important physiological role in liver-specific processes.

### Selenium treatment ameliorates toxicity induced by the chemotherapeutic agent cyclophosphamide *in vivo*

**Figure 5:**
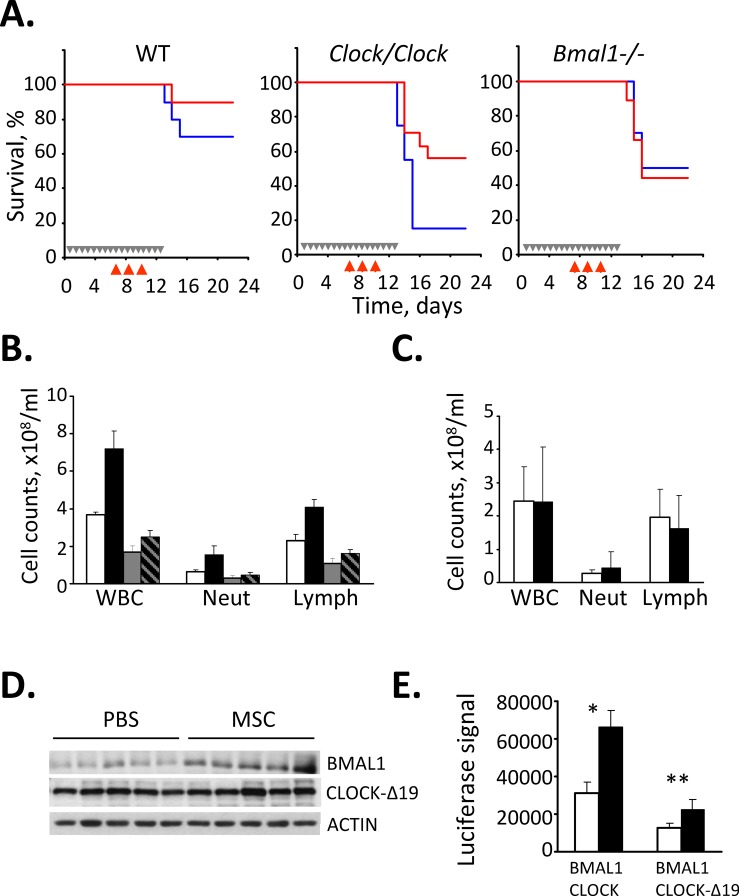
Selenium rescues cyclophosphamide-sensitive *Clock* mutant mice from drug-induced toxicity (A) Kaplan-Meyer survival curves. Wild type (WT), *Clock* mutant and *Bmal1−/−* mice were given either PBS (blue) or MSC (red, 7.5 mg/kg) daily for 2 weeks and injected with CY, 3x150mg/kg. Grey arrows in the Kaplan-Meyer plot indicate days of PBS or MSC administration; red arrows indicate days of CY injection. Treatment with MSC dramatically increased the 25-day survival rate of CY-treated *Clock/Clock* mice but had no effect on survival of *Bmal1* knockout animals. The experiment was performed three times with similar results. (B) Selenium treatment decreases CY-induced neutropenia and leukocytopenia. Total white blood cells (WBC), neutrophils (Neut) and lymphocyte (Lymph) counts in peripheral blood of wild type and *Clock* mutant mice treated with PBS or MSC and CY as described for Fig. 5A. MSC-induced increase in survival of CY-treated *Clock* mutant mice correlates with significant increase in the number of circulating lymphocytes (Student’s t-test p=0.015, n=5/group). (C) Selenium has no effect on the number of circulating lymphocytes in CY-treated *Bmal1−/−* mice. Mice were treated with PBS or MSC and CY as described in Fig. 5A. The cell count data are mean values for 8 mice. Please note that absolute values cannot be directly compared between the *Clock/Clock* (Fig. 4A) and *Bmal1−/−* mice since the experiments were not performed simultaneously. (D) Selenium administration increases BMAL1 protein abundance in the livers of *Clock/Clock* mice. Western blot analysis of liver extracts of five individual *Clock/Clock* mice fed with either PBS or MSC with anti-BMAL1 and anti-CLOCK antibodies. No effect on CLOCK-Δ19 expression level was detected. (E) Selenium increases CLOCK-Δ19/BMAL1-mediated activation of a responsive promoter. HEK293T cells were transfected with *Per1*-Luciferase reporter, HA-BMAL1 and either full-length wild type CLOCK or deletion mutant CLOCK (CLOCK-Δ19) and treated with indicated concentrations of MSA. Luciferase activity of cell lysates was normalized with β-Gal. Bars represent mean values ± standard error. Asterisk indicate statistically significant increase in promoter activation; *p=0.0011, **p=0.0012 (Student’s t-test).

Previously, we showed that sensitivity to the chemotherapeutic drug CY is modulated by the functional status of the CLOCK/BMAL1 transcriptional complex and that animals deficient in positive components of the molecular clock (*Clock* mutant and *Bmal1−/−* knockout mice) demonstrated enhanced sensitivity to CY-induced toxicity [15]. These data, together with the known ability of selenium to reduce chemotherapy-associated toxicity and our finding that selenium can modulate CLOCK/BMAL1 activity via up-regulation of BMAL1, prompted us to test whether selenium could rescue CY-sensitive circadian mutant mice from drug-induced toxicity. To test this hypothesis, we administered MSC to wild type, *Clock/Clock* mutant and *Bmal1−/−* knockout mice that received CY according to a previously described schedule [5, 15]. As expected, *Clock/Clock* and *Bmal1−/−* mice were significantly more sensitive to CY-induced toxicity manifested by severe hematopoietic syndrome. Selenium administration alleviated CY-induced toxicity in *Clock/Clock* mice, bringing both the survival rate (Fig. 5A) and number of circulating lymphocytes (Fig. 5B) to levels comparable to those of wild type animals. The rescuing effect correlated with an increase in BMAL1 protein levels in the livers of *Clock* mutant mice (Fig. 5D). In contrast, mice with genetic disruption of the *Bmal1* gene were non-responsive to rescuing effect of selenium as both control and MSC-treated *Bmal1−/−* mice showed similar kinetics of mortality and loss of circulating lymphocytes following the CY exposure (Fig. 5A,C) These data confirm that the rescuing effect of selenium *in vivo* is mediated, to a large extend, through BMAL1. The ability of selenium to increase CLOCK/BMAL1-dependent transactivation even when BMAL1 forms a complex with the mutant CLOCK protein expressed in *Clock/Clock* mice (CLOCK-Δ19) [34] was confirmed in cultured cells expressing ectopic BMAL1 and CLOCK-Δ19 (Fig. 5E). Together, these results suggest that selenium ameliorates CY-induced toxicity via its stimulatory effect on BMAL1 resulting in upregulation of CLOCK/BMAL1-mediated expression of target genes critical for survival/recovery of hematopoietic cells in the face of genotoxic stress.

## DISCUSSION

Cancer treatment involves frequent use of highly toxic compounds that commonly induce severe adverse effects reducing efficacy of therapy, creating risks of acquisition of additional diseases and reducing quality of life of cancer patients. That is why development of new therapeutic approaches allowing overcoming side effects of chemotherapy and radiation is highly desirable. In this respect, the components of the molecular clock are attractive therapeutic targets: they are ubiquitously expressed in virtually all tissues including those that determine an organism’s sensitivity to genotoxic treatments and more experimental evidence suggest that key circadian proteins can directly or indirectly modulate stress response pathways [17].

High-throughput screening of libraries of small organic molecules is one of the main tools for the discovery of bioactive compounds. This approach was successfully used recently to identify chemical compounds affecting basic circadian parameters (circadian period, amplitude and phase of rhythmicity) in circadian-synchronized cells [35, 36]. We reached similar goals using non-synchronized readout system that enabled selection of compounds modulating average level of activity of circadian transcriptional machinery revealed by the expression of the reporter driven by CLOCK/BMAL1-dependent circadian promoter. To our surprise, this screening approach identifies organic selenium compounds as potent up-regulators of *Per1* promoter.

Selenium is an essential trace element that has two major clinical applications: tumor prevention and protection against DNA damage induced by anti-cancer therapy. Studies in cell-based model systems as well as several clinical trials have conclusively demonstrated that selenium supplementation ameliorates radiation-induced mucositis in mice treated with fractionated doses of ionizing radiation [37] as well as radiation-induced diarrhea in treatment of patients with cervical and uterine cancers [38]. Our findings provide a plausible mechanism behind tissue protective effects of selenium by linking it to circadian regulation of gene expression. This study suggests that selenium is capable of tuning circadian transcriptional machinery to the higher activity associated with maximum resistance by upregulating BMAL1 expression.

Our data, as well as other reports [39, 40], show the effect of selenium to be highly tissue-specific. Thus, no changes in BMAL1 levels were detected in the SCN; accordingly, selenium administration has no effect on SCN-governed rhythms in locomotor activity. It is important to note that the lack of systemic effect of selenium on circadian rhythmicity presents an advantage from the therapeutic standpoint, as it allows for modulation of response to genotoxic treatments in a tissue-specific manner without disturbing the central clock.

Tissue specificity of the observed effects may also be the underlying reason for the selectivity of selenium-mediated protection for normal, as opposed to tumor, tissues. Testing of a large panel of tumor cell lines and animal tumor models would be needed to determine how universal the selectivity of BMAL1-mediated protective effects towards normal tissues is. Quite interestingly, recent reports demonstrated that in mammary gland, selenium administration induces upregulation of another circadian protein, PER2, in a BMAL1-independent manner [39, 40] and that this upregulation may underlie tumor preventive properties of selenium compounds. Thus, both cytoprotective and chemopreventive functions of selenium are likely to be exerted through the components of the molecular clock in a tissue-specific manner and presumably via different mechanisms.

Additional work is needed to fully understand the mechanism, by which the increase in CLOCK/BMAL1 activity ameliorates CY-induced toxicity. Our previous work has suggested that CLOCK/BMAL1-dependent modulation of the lymphocyte survival/recovery rate is an important factor in determining the *in vivo* drug response and survival in clinical therapy [15]. Consistent with this, studies with *Bmal1−/−* mice revealed the involvement of BMAL1 in differentiation of pre-B to mature B cells although direct molecular targets are still not known [41]. Another potential mechanism may involve BMAL1-dependent regulation of ROS homeostasis [42], which would protect against their excessive accumulation in response to genotoxic stress and thereby ameliorate drug-induced tissue damage. However, regardless of a precise molecular mechanism, the reported here ability of selenium to modulate activity of circadian transcriptional complex without affecting central clock, opens new possibilities for clinical applications. If previously we considered clock-targeting pharmaceuticals mostly as resetting agents (with the goal to reset molecular clocks in drug- and radiation-sensitive tissues to times of higher resistance to genotoxic treatment), now we have a compound capable of minimizing the damaging effects of genotoxic treatments by constant up regulation of circadian transcriptional activators in a tissue-specific manner. Since selenium is an essential trace element that is approved for clinical use it should be considered in clinical trials as chemo- and radiotherapy adjuvant.

## MATERIALS AND METHODS

### Chemicals

Library of Pharmacologically Active Compounds (LOPAC), cyclophosphamide (CY) and Se-Methyl-seleno-L-cysteine (MSC) were purchased from Sigma. Methyl seleninic acid (MSA) was purchased from PharmaSe Inc. (Lubbock, TX). Actonomycin D was purchased from Denville (Metuchen, NJ). DyLight594-conjugated donkey anti-Guinea pig IgG antibody was obtained from Jackson ImmunoResearch (West Grove, PA). Prolong Gold Antifade reagent containing DAPI was purchased from Invitrogen (Carlsbad, CA).

### Animals

Female *Clock* mutant mice [43-45] were backcrossed to C57BL/6J mice for 19 generations. Female *Bmal1* ^−/−^ mice [46] were backcrossed to C57BL/6J for 13 generations. Animals of 8-10 weeks old were synchronized to a 12h light: 12h dark cycle (LD 12:12) for at least 2 weeks before the experiment. Animals received selenium in the form of MSC either through a single i.p. injection at 10mg/kg or by gavage (once a day at ZT10) for 14 days at 7.5mg/kg daily [5]. For long-term experiments, selenium was administered with selenium supplemented diet (Harlan TD.01652; 3ppm of selenium) in the form of selenium selenite. Control animals received either regular (0.1ppm of selenium, TD.96363) or selenium-deficient diet (<0.01ppm; TD.92163). To assess acute effects of selenium on clock genes expression, tissue samples were collected 1 hr and 3 hrs after a single i.p. injection. In long-term experiments, tissue samples were collected after 14-days of MSC administration at two time points, ZT04 and ZT10. In all experiments tissue samples were immediately frozen in liquid nitrogen and stored at -80^0^ C until RNA and protein extraction. When MSC administration was combined with CY treatment, intraperitoneal injections of CY (150 mg/kg) were done at days 8, 10 and 12 as described previously [15]. CY-induced toxicity was assessed by mortality and body weight loss. The loss of 20% of the original body weight was considered an endpoint of the experiment. All animal studies were conducted in accordance with the regulations of the Committee on Animal Care and Use at the Roswell Park Cancer Institute.

### Plasmids and luciferase reporter constructs

Luciferase reporters containing the *mPer1* [47], *Bmal1* [31], *Cry1* [48] and *Per2* promoters [25] have been described previously. *Tieg1*-expressing construct is described in [32]. HA-*Bmal1*, HA-*Clock*, and HA-*ClockΔ19* are described in [48]. A specific TIEG1 binding site mutant of *Bmal1* promoter was generated by Site-directed mutageneis using QuikChange kit as per manufacturer’s recommendations (Stratagene, Inc., LaJolla, CA). The primer oligonucleotides used were 5’-CGATTGGTGttCGGGGttCCGGCCTG-3’ and its complement (lowercase italicized letter indicates substituted nucleotide).

### Cell-based assay optimization and library screening

Screening conditions were optimized by testing the assay for temporal and spatial signal variability using several 96-well plates as described in [18]. Luciferase readings of untreated cells (constant high luciferase signal) and cells treated with Actinomycin D (background signal) were used to calculate Z-factor using the equation:

$$Z=1-\frac{\left(3\delta s+3\delta c\right)}{\left|\mu s-\mu c\right|}$$

[49].

Absence of spatial (edge effects) and temporal artifacts and Z-factor between 0.6 and 0.8 validated the assay.

For screening, cells were plated onto the white clear-bottom 96-well tissue culture plates at 3x10^4^/well in DMEM supplemented with 10% FBS without phenol-red. JANUS automated liquid handling system (Perkin Elmer) equipped with pin tool (V&P Scientific, Inc) was used to deliver 100-120 nl of each of 1280 compounds present in LOPAC library to the 96-well assay plates. Luciferase activity was measured in 24-30 hrs after compound delivery using Bright-Glo Luciferase assay system and GloMax luminometer (Promega) according to manufacture’s protocol. Every compound that either reduced or increased the luciferase signal by more than 4 standard deviations was selected as a primary hit. Primary hits that reduced the luciferase signal were tested for general toxicity individually using CellTiter-Blue cell viability kit (Promega) according to manufacturer’s protocol.

### Transient transfection and luciferase reporter assay

HEK 293T cells were transiently transfected in 24-well plates with plasmid DNA (the final DNA amount was adjusted to 300 ng with pcDNA3 vector) using FuGene reagents (Roche) according to the manufacturer’s protocol. 10 ng of pcDNA3-βGal was included for normalization of transfection efficiency. Cells were collected for analysis 30h after transfection. Luciferase activity was measured with Luciferase Assay System (Promega, Madison, WI) according to manufacturer’s protocol. βGal activity was measured with 2-nitrophenyl-β-D-galactopyranoside as previously described [50].

### RNA isolation and real-time PCR analysis

Total RNA was isolated from cultured cells using RNeasy RNA extraction kit (Qiagen). Total RNA was isolated from livers using TRIZol reagent (Invitrogen) according to manufacturer’s protocol. TaqMan real-time RT-PCR was performed using the following ABI pre-made primers/probes sets:

*Clock*: Mm00455950-m1

*Bmal1*: Mm00500226-m1

*Per1*: Mm00501813-m1

*Per2*: Mm00478113-m1

*Cry1*: Mm00514392-m1

*Cry2*: Mm00546062-m1

Reactions were run on 7500 real-time PCR system (Applied Biosystems) and relative mRNA abundance was calculated using the comparative delta-Ct method with *Gapdh* mRNA as a standard as previously described [13].

### siRNA-mediated suppression of gene expression

Cells were seeded onto 24-well plates and transfected with siRNA against clock genes (siGENOME SMART pool, Dharmacon) according to manufacturer’s protocol. Reduction in protein levels was evaluated in tissue lysates collected 72 hrs post transfection by Western blotting with specific antibody. siGenome non-targeting siRNA was used as a negative control and Cyclophillin B siRNA was used as a positive control for efficiency of transfection.

### Western blot analysis and antibodies

Endogenous CLOCK and BMAL1 were detected with anti-CLOCK and anti-BMAL1 antibodies raised in guinea pig (Cocalico Biologicals, Inc., Reamstown, PA) according to the protocol described in [51]. Anti-actin antibody was purchased from Sigma (St.Louis, MO). HRP-conjugated secondary anti-rabbit, anti-mouse and anti-guinea pig antibodies were purchased form Jackson Laboratory.

### Immunohistochemisty

Deeply anesthetized animals were transcardially perfused with fixative (4% paraformaldehyde in PBS). Brains were removed immediately after perfusion and postfixed for 2 h in the same fixative at 4°C. After washing in PBS, brains were placed in 30% sucrose in PBS for cryoprotection, embedded in NEG -50 medium (Richard-Allan Scientific, Whaltham, MA), and sectioned at 12 μm. Coronal sections through the hypothalamus containing SCN were mounted on slides and processed for the immunohistochemical detection of BMAL protein using a standard immunofluorescence technique using primary antibody against BMAL1 at 1:100 dilution followed by secondary antibody staining (DyLight594-conjugated donkey anti-guinea pig IgG antibody, Jackson ImmunoResearch Laboratories, Inc., West Grove, PA) at 1:400 dilution. Sections were mounted with Prolong Gold Antifade reagent containing DAPI (Invitrogen, Carlsbad, CA) and analyzed with Axio Imager Z1 microsocpe (Zeiss) equipped with epifluorescence. Images were collected with Hamamatsu OTCA-R2 digital camera and AxioVision software.

### Behavioral analysis

Mice were singly housed in cages equipped with a running wheel at 12h light:12h dark cycle (LD12:12) for at least 2 weeks before being released into constant darkness (DD). Activity data were collected as previously described [43, 45]. The analyses were performed using ClockLab software (Actimetrics, Inc, Evanston, IL).

### Total Blood Cell Analysis

Hematological evaluation of blood composition was performed two days after the last CY injection. Peripheral blood obtained from the retroorbital sinus was collected into EDTA-treated tubes; the complete blood cells counts with WBC differentials were measured by using Advia 120 hematology system (Bayer) and analyzed with the software application for C57BL/6J mice. All control parameters were within the range previously described for this mouse strain.

### Statistical Analyses

Statistic significance in mRNA and protein levels was evaluated with Student’s t-test. P values <0.05 were considered statistically significant.

## Supplementary Figures and Tables


