# Supplementary material for: Selenium is a modulator of circadian clock that protects mice from the toxicity of a chemotherapeutic drug via upregulation of the core clock protein, BMAL1
Source: Oncotarget. 2011 Dec 31;2(12):1279–90. doi: 10.18632/oncotarget.411 (PMC3282084; doi:10.18632/oncotarget.411)
Supplement: Supplementary file 1 [file oncotarget-02-1279-s001.pdf]

Fig. S1The concentration of selenium in the diet of mice does not affect their circadian pattern of locomotor activity. Representative records of locomotor activity are shown for animals maintained on regular, selenium-free or selenium-enriched diets. Activity records are double plotted so that 48 hrs are represented horizontally. The black and white bar at the top represents the light schedule for the first 2 weeks of the experiment. Mice were synchronized to a light:dark cycle (LD 12:12) for 2 weeks before placement in constant darkness (DD) for the remainder of the experiment (days 14 through 35).

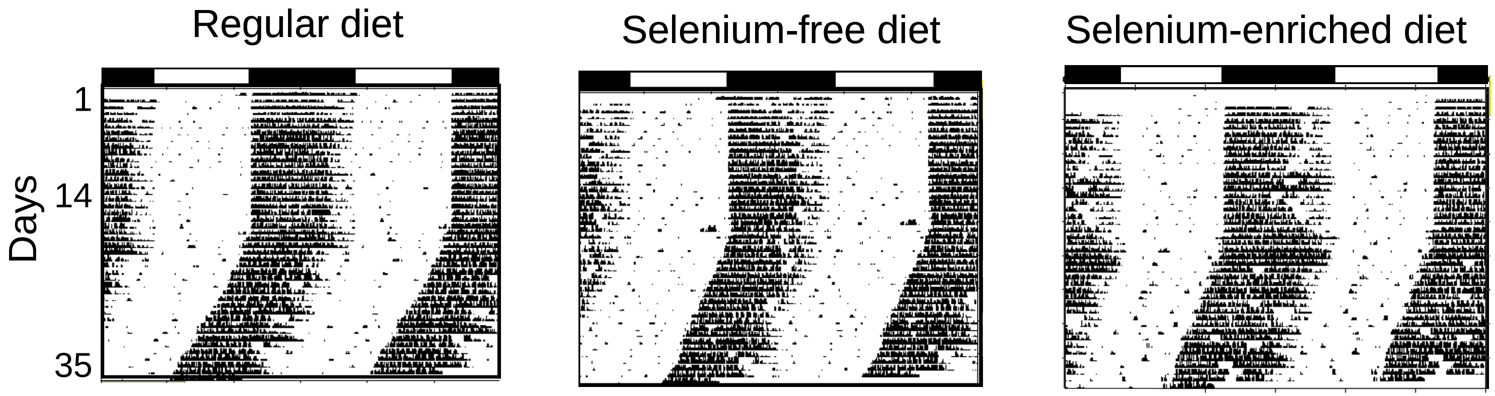

Table S1. Inhibitors of CLOCK/BMAL1-mediated activation of *Per1* promoter

Compound name	Function
Beclomethasone	Glucocorticoid
Budesonide	Glucocorticoid
Betamethasone	Glucocorticoid
Corticosterone	Glucocorticoid
Cyclosporin A	Calcineurin phosphatase inhibitor
SD-169	P38 MAPK inhibitor
Hydrocortisone	Glucocorticoid
Hydrocortisone 21-hemisuccinate sodium	Glucocorticoid
Ivermectin	Modulator of acetylcholine receptor
Mifepristone	Progesterone receptor antagonis
Cortexolone	Glucocorticoid
Rottlerin	PKC and CaM kinase III inhibitor
Triamcinolone	Glucocorticoid
Tyrphostin A9	PDGF tyrosine kinase receptor inhibitor

Table S2. Activators of CLOCK/BMAL1-mediated activation of *Per1* promoter

Compound name	Function
Cantharidine	Protein phosphatase 2A inhibitor
Colchicine	Inhibitor of tubulin polymerization
Cantharidic Acid	Protein phosphatase 1 (PP1) and 2A (PP2A) inhibitor
Forskolin	Activator of adenylate cyclase
Kenpaullone	Inhibitor of CDK
LY-294,002 hydrochloride	PI3K inhibitor
Kynurenic acid	Glutamate antagonist
Nocodazole	Disrupts microtubules by binding to beta-tubulin
2-methoxyestradiol	Estrogen
Norcantharidine	PP2A inhibitor
Se-(methyl)selenocysteine hydrochloride	Potent chemopreventive agent
Podophyllotoxin	Inhibitor of microtubule assembly
Quercetin	ATPase, PDE and PI3K inhibitor
Taxol	Inhibitor of tubulin disassembly
Vincristine sulfate	Inhibitor of microtubule assembly
Vinblastine sulfate salt	Inhibitor of microtubule assembly
(±)-Verapamil hydrochloride	Modulator of L-type Ca2+ Channel

Table S3. Parameters of circadian locomotor activity of animals on different selenium diet

	alpha time (hr)	rho time (hr)	tau (hr)	Total counts in DD (Rev/day)	Total counts in LD (Rev/day)
Regular diet	9.55±0.55	14.10±0.43	23.62±0.16	39469±6012	36165±5159
Selenium-free diet	9.32±0.86	14.25±0.86	23.57±0.11	33459±6866	31734±6383
Selenium-enriched diet	10.08±1.09	13.50±1.06	23.58±0.11	37557±5628	30967±5004
